# Combined High Dose-Rate Interstitial Brachytherapy and Stereotactic Body Radiotherapy in Unresectable Primary Vaginal Melanoma: A Case Report

**DOI:** 10.7759/cureus.109125

**Published:** 2026-05-18

**Authors:** Rojine T Ariani, Sabrina Khan, Puja Venkat

**Affiliations:** 1 Radiation Oncology, University of California Los Angeles David Geffen School of Medicine, Los Angeles, USA; 2 Dermatology, University of California Los Angeles David Geffen School of Medicine, Los Angeles, USA

**Keywords:** high dose rate interstitial brachytherapy, rare cancer of female genital tract, rare mucosal malignancy, sbrt (stereotactic body radiotherapy), vaginal melanoma

## Abstract

Primary vaginal melanoma is a rare and aggressive malignancy with limited prospective data to guide optimal management, particularly in unresectable disease. This report describes a 40-year-old woman with unresectable, locally progressive primary vaginal melanoma refractory to first-line combination immune checkpoint inhibition. She was treated with a dose-escalated, multimodality radiotherapy approach incorporating high-dose-rate interstitial brachytherapy to the primary tumor and stereotactic body radiotherapy with simultaneous integrated boost to nodal disease. Treatment was well tolerated and resulted in a complete metabolic and radiographic response at one year. This case highlights the role of integrated, hypofractionated radiotherapy strategies in achieving durable locoregional control in patients with progressive disease despite systemic therapy.

## Introduction

Primary vaginal melanoma (PVM) is a rare and aggressive gynecological malignancy, accounting for less than 3% of vaginal cancers and approximately 1% of all melanomas [1,2]. Prognosis remains poor, driven by delayed diagnosis, early dissemination, and limited responsiveness to therapy. For unresectable disease, current management centers on combination immune checkpoint inhibition (ICI), with radiotherapy (RT) used selectively for locoregional control. However, the optimal role and integration of RT remain undefined due to both the rarity of PVM and the historically limited perceived responsiveness of melanoma to conventional radiation approaches [3]. Increasing experience with hypofractionated and dose-escalated RT strategies has supported RT as a potentially effective modality for locoregional disease control. High-dose-rate interstitial brachytherapy (HDR-ISBT) permits conformal focal dose escalation through catheter-based placement of the radioactive source directly within or adjacent to tumor tissue, while stereotactic body RT (SBRT) enables highly conformal delivery of ablative external-beam doses to discrete targets. This case report highlights the role of a dose-escalated, multimodality RT strategy incorporating HDR-ISBT and SBRT in a patient with unresectable, locally progressive PVM refractory to first-line combination ICI. Written informed consent for treatment was obtained from the patient.

## Case presentation

A 40-year-old woman presented with five months of irregular vaginal bleeding, urinary frequency, and urinary leakage. Pelvic exam revealed a palpable tumor involving the posterior one-third of the vagina with extension along the lower to mid right vaginal wall, approaching the vulva. Staging with CT chest, abdomen, and pelvis (C/A/P), Positron Emission Tomography-Computed Tomography (PET-CT), and brain MRI demonstrated a heterogeneous vaginal mass involving the cervix and right vaginal canal measuring up to 5.5 cm, with fluorodeoxyglucose (FDG)-avid right inguinal and left pelvic lymphadenopathy and a solitary punctate enhancing right frontal lobe lesion. Given the tumor extent and nodal involvement, the disease was deemed unresectable. Pathology from vaginal wall biopsy was initially misinterpreted as poorly differentiated invasive squamous cell carcinoma (SCC), with plans for cisplatin and pembrolizumab. Pathologic re-review confirmed malignant melanoma with S100 and SOX10 positivity, PD-L1 expression of 10% with a combined positive score of 15, human papillomavirus (HPV) negativity, and wild-type p53. Accordingly, the treatment plan was revised to a combination of nivolumab and ipilimumab, with interval imaging planned to reassess resectability after four treatment cycles.

Despite resolution of the intracranial lesion, CT antero-posterior (A/P) demonstrated progression of the primary vaginal tumor to approximately 10.4 cm with a mixed nodal response. Given the limited systemic response with ongoing symptomatic local progression, definitive RT was pursued for local control, with continuation of maintenance nivolumab. Pre-RT pelvic MRI demonstrated a lobulated vaginal mass measuring 9.2 cm with near circumferential mid-vaginal involvement, introital extension, abutment, and possible invasion of the posterior anal sphincter, anterior abutment of the urethra, and enlarged metastatic inguinal lymph nodes (Figure 1).

**Figure 1:**
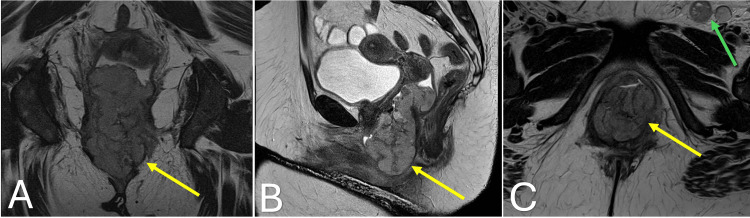
Pre-radiotherapy MRI pelvis Pre-radiotherapy MRI pelvis demonstrating the large primary vaginal tumor on (A) coronal, (B) sagittal, and (C) axial views (yellow arrow). Representative pathologically enlarged left inguinal lymph nodes (green arrow) are also visualized on the axial view (C).

For the primary tumor, she underwent image-guided High-Dose-Rate Interstitial Brachytherapy (HDR-ISBT) using a combined intracavitary and interstitial technique to a dose of 30 Gy in 3 fractions delivered in 3 implants over 2 weeks, yielding a primary tumor equivalent dose in 2 fractions (EQD2) of approximately 83 Gy (α/β = 2.5). Examination under anesthesia demonstrated a large posterior vaginal tumor protruding from the introitus with rectovaginal septum involvement and a discrete right vulvar nodule. A modified Syed interstitial implant was used, with insertion of 17 interstitial catheters under transrectal ultrasound guidance, and an additional tube-and-button implant using two parallel catheters for the vulvar lesion to safely bring the dose to the skin surface (Figure 2). Concurrent Stereotactic Body Radiation Therapy (SBRT) was delivered using volumetric modulated arc therapy (VMAT) to 25 Gy in 5 fractions to the elective nodal volume covering the bilateral inguinal, pelvic, and paraaortic nodes, with a simultaneous integrated boost (SIB) to 40 Gy in 5 fractions to gross nodal disease (Figure 3). A 5mm uniform expansion of the CTV was applied to form the planning target volume (PTV).

**Figure 2:**
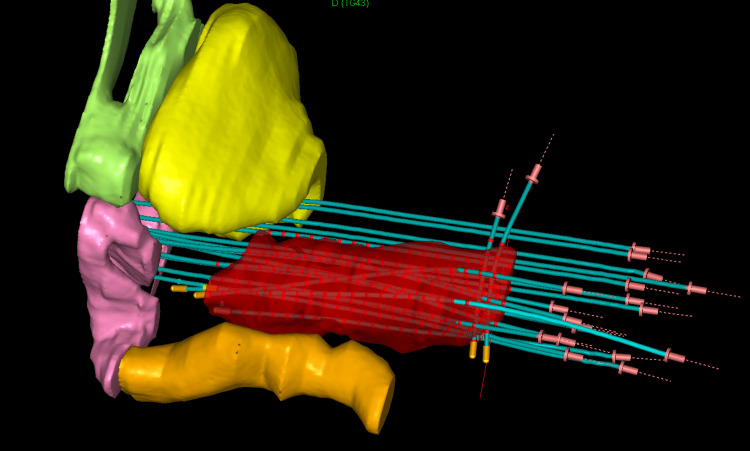
Three-dimensional treatment reconstruction of the combined intracavitary and interstitial HDR brachytherapy implant. A modified Syed-based interstitial implant with 17 transperineal catheters was used to encompass the bulky posterior vaginal and rectovaginal septal disease. In addition, a tube-and-button implant with two parallel catheters was placed to treat the discrete vulvar lesion and facilitate dose delivery to the skin surface. The reconstruction demonstrates the spatial geometry and catheter distribution used to achieve target coverage while respecting adjacent pelvic organs at risk, including the bladder (yellow structure) and rectum (orange structure).

**Figure 3:**
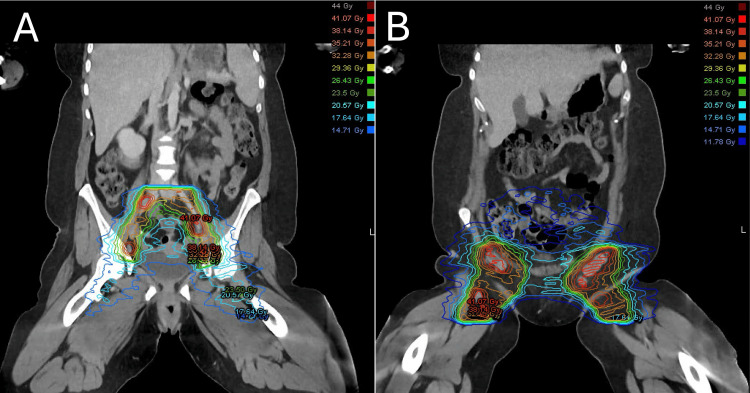
Isodose line view of stereotactic body radiotherapy plan with simultaneous integrated boost to lymph nodes Isodose line view of the stereotactic body radiotherapy plan delivering 30 Gy in 5 fractions to the elective nodal volume encompassing the bilateral inguinal, paaaortic, and pelvic nodes, with a simultaneous integrated boost to 40 Gy in 5 fractions to gross nodal disease, shown in the coronal plane demonstrating (A) paraaortic and pelvic nodal coverage and (B) inguinal nodal coverage.

Composite Equivalent Dose in 2 Gy fractions (EQD2) summation, using an α/β ratio of 3, was performed to evaluate the cumulative dose contributions from both the HDR-ISBT and SBRT courses to organs at risk (OARs), including the bladder, bowel, sigmoid colon, and rectum. Dose to OARs was assessed using D0.1cc and D2cc metrics. Composite cumulative EQD2 D0.1cc values were 92.12 Gy for bladder, 53.35 Gy for bowel, 68.12 Gy for sigmoid, and 75.46 Gy for rectum. Composite cumulative EQD2 D2cc values were 73.06 Gy for bladder, 48.73 Gy for bowel, 55.11 Gy for sigmoid, and 61.59 Gy for rectum.

Early follow-up demonstrated good tolerance of therapy, with minimal vaginal bleeding that resolved spontaneously. By three months post-treatment, restaging PET/CT and pelvic MRI showed no evidence of disease (Figure 4). By that time, she had developed vaginal stenosis requiring ongoing dilator use and mild urinary incontinence managed conservatively. One-year post-treatment PET/CT continued to demonstrate no evidence of disease, with no additional late toxicities observed.

**Figure 4:**
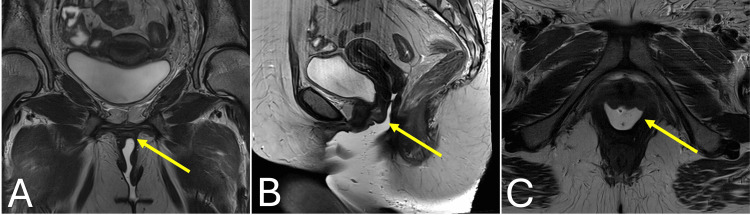
Three months post-radiotherapy MRI pelvis Three months post-radiotherapy MRI pelvis demonstrating complete resolution disease on (A) coronal, (B) sagittal, and (C) axial views.

## Discussion

PVM is an aggressive mucosal melanoma characterized by high rates of local recurrence and distant metastasis, with a reported 5-year overall survival below 20% [2]. Outcomes are limited in part by delayed diagnosis, as lesions are often clinically nonspecific and may be amelanotic, leading to frequent misidentification as more common gynecologic malignancies [2,4,5]. This diagnostic challenge was evident in our patient case, where the tumor was initially misdiagnosed as squamous cell carcinoma, reflecting the nonspecific clinical presentation and accompanying lack of initial suspicion for melanoma. On re-interpretation utilizing melanoma-specific tumor markers, she was correctly diagnosed with PVM and treated accordingly, underscoring the importance of incorporating markers including SOX10, Melan-A, HMB45, and Vimentin when evaluating vulvovaginal lesions in reaching a correct diagnosis [4-6]. Notably, although PVM typically occurs in post-menopausal patients, our presented case highlights that younger individuals may also present with advanced disease, reinforcing the need for vigilance in evaluation across age groups [2].

Systemic therapy for unresectable PVM is centered on combination ICI, most commonly with ipilimumab and nivolumab [3]. This approach has demonstrated greater efficacy than single-agent therapy, with objective response rates of 40.9% versus 23.3%, respectively [7]. Nonetheless, outcomes for mucosal melanoma, including PVM, remain inferior to those observed in cutaneous melanoma, with objective response rates of 37.1% versus 60.4% for cutaneous melanoma, respectively [7]. In our patient, treatment resulted in a complete intracranial response but concurrent progression of the primary tumor and mixed nodal response, reflecting the variable sensitivity of mucosal melanoma to ICI and the need for additional local therapy in selected patients.

RT plays a key role in this setting, particularly for patients with persistent or progressive locoregional disease despite optimal systemic therapy. Although guidelines support its use with or without immunotherapy, no standardized approach to technique, dose, or sequencing exists, given the rarity of PVM and the absence of prospective data [3]. Current recommendations favor definitive treatment with external-beam radiation therapy (EBRT) and/or brachytherapy with dose escalation to the primary tumor and involved regional nodes, reflecting the need for aggressive local therapy in this disease context [3,8-10].

In our patient, a multimodality, dose-escalated hypofractionated approach was selected to reflect the radiobiologic characteristics of melanoma and optimize local control. The primary tumor was treated with image-guided HDR-ISBT using a template-based modified Syed technique with transrectal ultrasound guidance, allowing conformal coverage of extensive disease, including vulvar extension. Regional nodal disease was treated concurrently with Volumetric Modulated Arc Therapy (VMAT)-based SBRT, inclusive of SIB, to gross nodal metastases. Together, this integrated strategy provided comprehensive treatment of all active disease sites within a condensed course.

Composite cumulative EQD2 analysis (α/β=3) of OARs demonstrated bladder and rectum D2cc​ values of 73.06 Gy and 61.59 Gy, respectively. Although disease-specific dose constraints for PVM have not been established, these values remain comparable to published image-guided gynecologic brachytherapy tolerance parameters, such as the EMBRACE II recommendations targeting cumulative rectal D2cc​≤65 Gy to minimize late toxicity [11,12]. Importantly, the precision of HDR-ISBT permitted substantial focal dose escalation while maintaining this acceptable OAR exposure, a key technical advantage given the relative radioresistance of mucosal melanoma and evidence supporting improved control with larger dose per fraction and intensified radiation delivery [10,13,14].

Early post-treatment imaging demonstrated a complete metabolic and radiographic response, which remained durable at one year following treatment completion. Treatment was well tolerated, with late toxicity limited to expected vaginal stenosis managed conservatively. These favorable oncologic and toxicity outcomes align with limited published data supporting the use of definitive RT for PVM [9,15,16]. Inagaki et al. reported a complete response maintained at 15 months following HDR brachytherapy (48 Gy in 8 fractions) for PVM with urethral invasion, highlighting that the low α/β ratio of melanoma (approximately 2.5) provides a strong radiobiologic rationale for such hypofractionated approaches [9]. Similarly, Orellana et al. reported durable local control exceeding five years after EBRT followed by an HDR boost, totaling a cumulative primary tumor EQD2 of approximately 70 Gy [16]. By comparison, our approach delivered a cumulative primary tumor EQD2 of 83.3 Gy (α/β=2.5), effectively leveraging a 10 Gy per fraction schedule to overcome inherent radioresistance.

Collectively, these findings support the role of individualized, dose-escalated multimodality RT in achieving meaningful locoregional control for unresectable PVM, particularly in patients with heterogeneous responses to systemic immunotherapy. The role of the tumor microenvironment (TME) in contributing to this complete response also warrants consideration, particularly given the heterogeneous response observed following initial ICI [8]. High-dose RT to the primary tumor and regional nodal disease may have altered the local immune milieu in a manner that contributed to subsequent disease control. Although paired pre- and post-treatment biopsy specimens were not available in our case to evaluate dynamic TME changes following ICI and RT, the durable complete response observed at one-year post-treatment suggests a potential interaction between focal radiation and the underlying immune microenvironment. Although a longer follow-up is necessary to characterize long-term durability, this case adds to the growing evidence supporting aggressive local therapy in this rare and challenging disease entity.

## Conclusions

Management of PVM remains challenging due to its rarity and the absence of standardized treatment paradigms. Although the combination ICI constitutes the backbone of therapy for unresectable disease, responses are frequently heterogeneous and may fail to provide adequate local control. This case demonstrates that a tailored, dose-escalated RT approach incorporating HDR-ISBT and SBRT with SIB can effectively treat progressive primary and nodal disease, resulting in complete response with minimal toxicity and ongoing disease-free status at one year following treatment completion. While conclusions from a single-case experience remain inherently limited, these findings support the potential role of individualized, multimodality RT strategies in select patients, particularly when systemic therapy alone is insufficient. Given the aggressive biology and potential for delayed recurrence in primary vaginal melanoma, longer follow-up will be important to further characterize the durability of response and long-term toxicity profile associated with this treatment strategy. Continued accumulation of clinical experience and prospective investigation will be essential to refine treatment approaches and improve outcomes in this population.
